# A Complex Clinical Situation in Polycystic Ovary Syndrome: HAIR-AN Syndrome ‘‘Case Report”

**DOI:** 10.1155/carm/5825601

**Published:** 2025-04-29

**Authors:** Ramin Alizadeh Gheshlagh, Senay Topsakal

**Affiliations:** ^1^Faculty of Medicine, Pamukkale University, Denizli, Türkiye; ^2^Faculty of Medicine, Department of Endocrinology and Metabolism, Pamukkale University, Denizli, Türkiye

**Keywords:** acanthosis nigricans, case report, HAIR-AN, hyperandrogenism, severe insulin resistance

## Abstract

Hyperandrogenism, insulin resistance, and acanthosis nigricans (HAIR-AN) syndrome is a distinct and uncommon form of polycystic ovarian syndrome. It manifests through hyperandrogenism (HA), insulin resistance (IR), and acanthosis nigricans (AN), along with symptoms like acne, hirsutism, irregular menstruation, and other androgen-related issues. A 17-year-old female with a history of childhood obesity and irregular menstrual cycles presented with weight gain and amenorrhea. Previously assessed for hirsutism with a Ferriman–Gallwey score of 14, she was found to have hepatic steatosis, ovarian cysts, and IR. She was advised to lose weight and prescribed metformin but did not adhere to the treatment. Four years later, she returned with further weight gain and hirsutism and was diagnosed with androgenetic alopecia. The presence of AN, HA, and severe IR led to a diagnosis of HAIR-AN syndrome, and she was placed under observation. We used next-generation sequencing (NGS) to screen 70 genes for mutation and identify relevant genetic variations. The investigation targeted all exons and exon-intron junctions in genes, including ACOX1, GM2A, ACSF3, and others. Bioinformatics tools and in silico algorithms were used to assess the impact of the variants. No significant mutations associated with the patient's symptoms were identified. HAIR-AN syndrome can present in various forms and should be considered in cases of unexplained AN and menstrual irregularities. Early detection, diagnosis, and treatment of HAIR-AN syndrome can alleviate symptoms and improve patients' quality of life. This case presentation aims to evaluate the findings of a HAIR-AN syndrome that became very severe due to treatment noncompliance.

## 1. Introduction

Polycystic ovary syndrome (PCOS) encompasses a range of clinical manifestations, with one distinct variation being hyperandrogenism, insulin resistance, and acanthosis nigricans (HAIR-AN) syndrome. This syndrome is characterized by a triad of clinical features: hyperandrogenism (HA), insulin resistance (IR), and acanthosis nigricans (AN). It primarily affects women and can present with symptoms such as acne, hirsutism, irregular menstruation, and other androgen-related issues. The relationship between HA and IR plays a central role in the pathophysiology of HAIR-AN syndrome, with each component exacerbating the other, thus creating a vicious cycle of hormonal and metabolic disturbances [1–3].

HAIR-AN syndrome is considered a specific subphenotype of PCOS, distinguished by more severe manifestations of IR and hyperinsulinemia. Women with HAIR-AN syndrome tend to experience more severe IR compared to those with typical PCOS, with IR often linked to adipose tissue dysfunction, marked by elevated leptin levels and decreased adiponectin. AN, a condition that manifests as dark, velvety patches of skin, serves as a key clinical feature of HAIR-AN but is less common in other forms of PCOS. Obesity is also more prevalent and severe in HAIR-AN syndrome, which further contributes to IR and metabolic dysfunction in these individuals [3–5].

The prevalence of HAIR-AN syndrome is estimated to be around 5% in female adolescents, with the syndrome often emerging during adolescence or early adulthood, a period of significant hormonal changes. Its occurrence is notably higher in specific ethnic groups, including African American, Hispanic, and South Asian women, who are also at an increased risk for metabolic disorders such as IR and type 2 diabetes. Additionally, a family history of PCOS, type 2 diabetes, IR, or HA increases the likelihood of developing HAIR-AN syndrome, suggesting a genetic predisposition [6–8].

Diagnosis of HAIR-AN syndrome involves a combination of clinical evaluation and laboratory tests. Key markers include AN, HA, fasting insulin and glucose levels, the oral glucose tolerance test (OGTT), and the hyperinsulinemic-euglycemic clamp method. IR leads to hyperinsulinemia, which in turn stimulates the ovaries to produce androgens, exacerbating HA. Symptoms of severe HA are common in HAIR-AN syndrome and often include significant hirsutism, alopecia, and acne [8, 9].

Furthermore, IR in HAIR-AN is a primary contributor to obesity, type 2 diabetes, and metabolic syndrome. AN, a visible marker of underlying IR, typically appears in areas like the neck, armpits, and groin. The syndrome highlights the importance of early identification and management to prevent long-term complications, with treatment strategies including lifestyle modifications, weight management, and pharmacological interventions such as insulin-sensitizing agents. However, patient adherence to these treatments is often challenging, contributing to worsening symptoms and progression of the syndrome [7–9].

A 17-year-old girl with a history of childhood obesity and irregular menstrual cycles who was diagnosed with HAIR-AN syndrome is the subject of this case. Despite receiving initial medical advice and prescribed treatments, noncompliance led to an exacerbation of her condition. This case underscores the importance of early detection, consistent management, and patient compliance in controlling HAIR-AN syndrome.

## 2. Case Report

The patient, a 17-year-old female with a history of childhood obesity and irregular menstrual cycles, presented with complaints of weight gain and amenorrhea. She had menarche at age 12, followed by the development of hirsutism, with a Ferriman–Gallwey score of 14, indicating significant HA. Abdominal–pelvic ultrasound revealed normal ovarian sizes (right ovary: 20 × 25 × 29 mm, left ovary: 18 × 22 × 30 mm), and while multiple scattered follicular cysts were observed, they did not suggest polycystic ovarian morphology typical of PCOS. The largest cyst measured 5 mm. Hepatic steatosis was also noted, raising concerns about IR.

The diagnosis of IR was confirmed through laboratory testing. The patient's fasting insulin levels were elevated at 84.9 µIU/mL (2017), well above the reference range (2.6–24.9 µIU/mL). Using the Homeostasis Model Assessment of Insulin Resistance (HOMA-IR), which is based on fasting insulin and glucose levels, the patient's HOMA-IR was calculated at 18.46, indicating severe IR (normal range: < 2.0). An OGTT was also performed, confirming the diagnosis of IR, although specific glucose values during the test were not provided.

**Figure 1:**
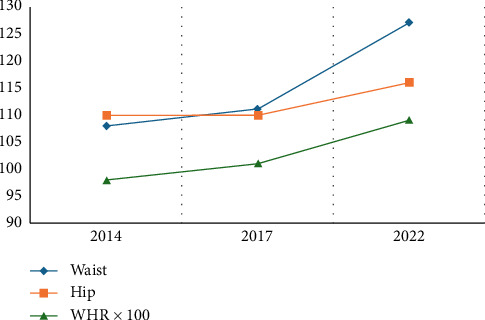
Monitoring of waist and hip measurements.

The patient had a history of significant weight gain, from 84 kg in 2017 to 118 kg in 2022, accompanied by an increase in BMI (33.3–40.4 kg/m^2^) and waist-to-hip ratio (0.98–1.09) (Table 1 and Figure 1). Laboratory findings revealed persistent dyslipidemia, with triglyceride levels increasing from 212 mg/dL (2008) to 288 mg/dL (2022), and low HDL cholesterol levels (39 mg/dL). Liver enzymes remained elevated, with ALT levels reaching 71 IU/L (reference: 19–25 IU/L) and GGT levels increasing to 60 U/L (reference: < 30 U/L). In addition, elevated levels of dehydroepiandrosterone sulfate (DHEAS) were noted, peaking at 460 μg/dL in 2014 and 405 μg/dL in 2017, with SHBG levels consistently low (as low as 12.4 nmol/L in 2011) (Table 2).

**Figure 2:**
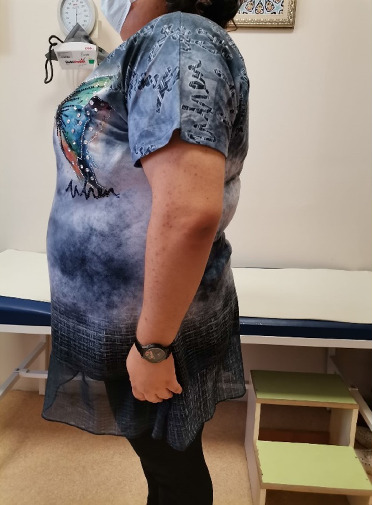
Truncal obesity.

**Figure 3:**
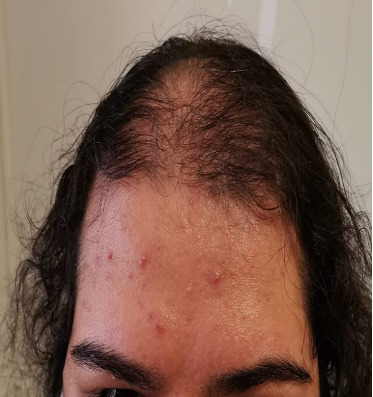
Androgenetic alopecia.

**Figure 4:**
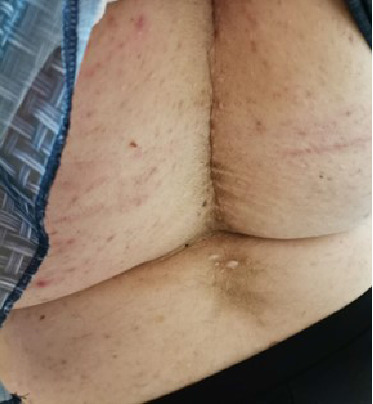
Widespread skin tags on the back.

**Figure 5:**
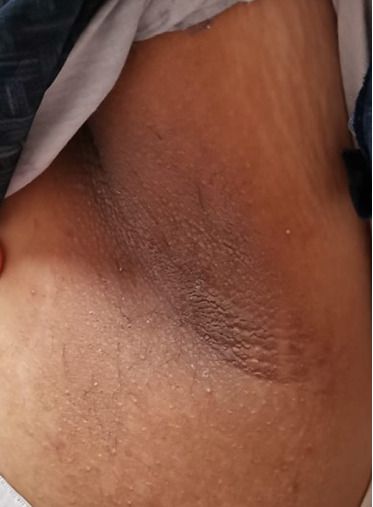
Acanthosis nigricans.

Four years after her initial diagnosis, the patient returned with worsened symptoms of weight gain and hirsutism. Follow-up examination revealed worsening of HA, including the development of androgenetic alopecia, truncal obesity, widespread hirsutism, and AN. These clinical features, in conjunction with severe IR, led to the diagnosis of HAIR-AN syndrome, a rare variant of PCOS characterized by the triad of HA, IR, and AN (Figures 2, 3, 4, and 5).

Despite being advised to lose weight and being prescribed metformin, the patient did not adhere to the recommended treatment regimen, leading to the progression of her condition. The patient consented to the use of her data for research purposes, and informed consent was obtained.

Mutation screening of 70 genes, conducted using next-generation DNA sequencing (NGS), revealed no significant mutations associated with the patient's symptoms. The screening included genes associated with various metabolic and genetic disorders, including ACOX1, GM2A, and several others. In silico prediction algorithms and online databases were utilized to interpret the results, with no pathogenic mutations identified.

Overall, this case underscores the importance of early detection, adherence to treatment, and the need for careful monitoring of metabolic parameters in managing HAIR-AN syndrome.

## 3. Treatment

Lifestyle modifications, including dietary changes, physical activity, behavioral adjustments, alcohol consumption, smoking cessation, sleep management, psychological support, vitamins, minerals, supplements, herbal remedies, acupuncture, yoga, and other complementary therapies, are crucial for the management of HAIR-AN syndrome. These interventions were discussed with the patient, and metformin (500 mg twice daily) was prescribed as part of the treatment regimen. However, the patient did not adhere to the prescribed therapy. Subsequent recommendations included pioglitazone (30 mg once daily) and liraglutide (1.2 mg/day), but the patient declined both treatments and was followed up irregularly. Like many individuals with HAIR-AN syndrome, the patient's failure to comply with treatment and inability to lose weight resulted in the worsening of her metabolic condition. Despite being informed about the potential benefits of psychiatric support, the patient refused this intervention. This case underscores the progression of the disease in an untreated patient and highlights the significance of integrating psychiatric support into the management of this chronic condition, which affects various facets of the patient's life, including anxiety, depression, psychosexual dysfunction, eating disorders, negative body image, and poor quality of life (QoL). The incorporation of psychiatric support is vital, as it can enhance psychological well-being, reduce stress, and improve treatment adherence [10].

## 4. Discussion

HAIR-AN syndrome is a rare and subphenotype of PCOS characterized by marked IR and hyperinsulinemia, often attributed the defects in insulin receptor genes [3]. Elevated insulin levels can directly stimulate the overproduction of androgens in the ovaries which exacerbates the symptoms of HA [6]. In adolescent patients, HAIR-AN syndrome may lead to menstrual irregularities, hyperandrogenic manifestations, and IR. As previously discussed, HAIR-AN syndrome is considered a specific subphenotype of PCOS, with distinct clinical and pathophysiological features, including IR, hyperinsulinemia, and HA, although the severity and appearance of these features vary. Women with HAIR-AN syndrome exhibit more severe IR and hyperinsulinemia compared to those with PCOS. Severe IR in HAIR-AN syndrome is often associated with adipose tissue dysfunction, characterized by high leptin levels and decreased adiponectin. AN, which manifests as dark, velvety patches of skin, is a hallmark feature of HAIR-AN syndrome, although it is less common in PCOS. Furthermore, obesity is more prevalent and severe in HAIR-AN syndrome, contributing to exacerbated IR in these patients [5, 8]. Management approaches also differ: While HAIR-AN management focuses on addressing IR through medications such as liraglutide and lifestyle modifications, hormonal contraception for cycle regulation and metabolic symptom management are typical treatments for PCOS. HAIR-AN syndrome is estimated to affect approximately 5% of female adolescents in clinical settings [2].

The differential diagnosis of adolescent HA is broad and includes a wide range of conditions such as congenital adrenal hyperplasia, gonadal dysgenesis, Cushing syndrome, tumors, and glucocorticoid resistance. Therefore, before diagnosing HAIR-AN syndrome, these alternative conditions must be excluded [7, 8]. This case presents a challenging clinical scenario of HAIR-AN syndrome in a 17-year-old female with a history of childhood obesity and irregular menstrual cycles. She exhibited several characteristic features of HAIR-AN syndrome, including HA, severe IR, and AN. Additionally, clinical manifestations such as hirsutism, androgenetic alopecia, and persistent metabolic disorders were observed.

This case highlights the challenges in treating HAIR-AN syndrome, particularly with the development of weight gain and worsening metabolic parameters. IR, often linked to mutations in the insulin receptor gene, is considered the primary issue in these patients. Elevated insulin levels promote the action of LH on granulosa cell steroidogenesis, thereby stimulating androgen synthesis and ovarian steroidogenesis. Insulin also interacts with insulin-like growth factor receptors, which further increases ovarian androgen synthesis [8–12]. Although PCOS is similarly characterized by HA, ovulatory dysfunction, and polycystic ovarian morphology, leading to infertility, the severity of these features is generally more pronounced in HAIR-AN syndrome. It disrupts regular ovulation, which means eggs may not be released regularly due to hormonal imbalances, particularly elevated androgens and IR [13, 14]. In fact, IR is considered intrinsic to PCOS, contributing to reproductive morbidity, including infertility, spontaneous abortion, and complications like gestational diabetes [15]. Studies suggest that treatments like clomiphene citrate or letrozole may achieve cumulative live birth rates of around 70% in PCOS patients. Lifestyle interventions, including weight loss, are also crucial. Even a modest 5%–10% reduction in body weight can help restore ovulatory cycles and improve pregnancy rates. For patients unresponsive to other treatments, in vitro fertilization (IVF) may be considered as a treatment option [16–21].

In HAIR-AN syndrome, IR is more severe than standard PCOS, which may exacerbate fertility challenges. This suggests that fertility in HAIR-AN syndrome may require more intensive interventions, including the combination of metformin with ovulation induction therapies [5, 8, 22]. Persistent dyslipidemia, evidenced by elevated triglycerides, low HDL levels, and borderline high LDL levels, is common in these patients and increases their cardiovascular risk in these patients. Women with HA are at a higher risk of hyperlipidemia and secondary coronary artery disease, which underscores the need for regular lipid monitoring in this patient group [23, 24].

PCOS and HAIR-AN are associated with increased cardiovascular risk and poor cardiovascular outcomes. Some studies show that bariatric surgery reduced cardiovascular mortality, making it a potential treatment option for women with class 2 obesity (BMI 35–40 kg/m^2^) [25, 26]. Women with PCOS have a significantly higher risk of cardiovascular events due to IR and increased adipose tissue lipolysis, which lead to dyslipidemia and vasoconstriction.

Hyperinsulinemia can also cause sympathoexcitation, resulting in greater renal water retention and elevated blood pressure. Moreover, PCOS is linked to endothelial dysfunction, arterial stiffness, and increased carotid intima-media thickness [27–38]. Persistent high ALT and GGT levels indicate liver involvement, likely related to hepatic steatosis, which is commonly associated with obesity and IR [23, 24]. The findings in this case align with these observations, suggesting similar underlying mechanisms.

The absence of significant genetic abnormalities in the examined genes in our patient suggests that HAIR-AN syndrome is primarily influenced by environmental and lifestyle factors rather than a genetic predisposition. Increasing awareness of this syndrome and providing multidisciplinary care, including obstetrics, psychiatry, and nutrition, are essential steps in managing the condition. One of the key challenges is ensuring patient adherence to lifestyle modification, including diet, physical activity, and psychological support, all of which are critical for managing the disease. However, as seen in this case, many patients either refuse or fail to implement the recommended treatment. Psychological issues such as anxiety, depression, psychosexual dysfunction, eating disorders, and poor body image are common in HAIR-AN patients, which often contributes to poor treatment compliance. Addressing these psychological issues through psychiatric support is essential to improving patient outcomes and enhancing adherence to treatment [9].

Treatment options for hirsutism include the 5-alpha reductase inhibitor flutamide, the androgen receptor antagonist spironolactone, and estrogen-progestin oral contraceptives, all of which have been shown to be effective in reducing symptoms. Metformin, an insulin sensitizer, is commonly used to address metabolic abnormalities in HAIR-AN patients. Recent case reports suggest that the glucagon-like peptide 1 (GLP-1) receptor agonist liraglutide may improve menstrual regularity and metabolic profiles. Additionally, bariatric surgery, such as sleeve gastrectomy, has shown promise in improving insulin sensitivity and promoting weight loss in HAIR-AN patients, although it may not fully normalize testosterone levels. In selecting a treatment plan, factors such as the severity of the condition, patient preferences, and risk tolerance must be considered [35–38].

Despite repeated diet therapy, this patient failed to lose weight, did not respond to metformin, and refused other recommended treatments. She frequently neglected follow-ups, resulting in a decline in her overall health and QoL. Studies show that both PCOS and HAIR-AN syndrome significantly affect patients' QoL and psychological well-being. This impacts treatment adherence, highlighting the importance of psychiatric support in managing these conditions [29].

Delayed treatment of HAIR-AN syndrome can lead to the progression of metabolic disorders such as IR, HA, and obesity, ultimately increasing the risk for type 2 diabetes, cardiovascular diseases, and infertility. Delayed diagnosis can also contribute to masculinization symptoms, such as hirsutism, and exacerbate psychological problems, including depression and anxiety. Early diagnosis and intervention can reduce treatment costs, prevent the progression of the syndrome, and improve patient outcomes. Delayed treatment may complicate the treatment process, often necessitating more invasive interventions and leading to a worsened prognosis and reduced QoL.

Environmental factors, such as a sedentary lifestyle, poor diet, and chronic stress, play a significant role in the onset and progression of HAIR-AN syndrome. These factors can exacerbate IR and contribute to the development of obesity, further aggravating the condition. Additionally, endocrine disruptors—chemicals that interfere with hormonal regulation—can negatively affect hormone balance, which may contribute to the development of HAIR-AN syndrome [39–41].

## 5. Conclusion

This case highlights the importance of early diagnosis and comprehensive management of HAIR-AN syndrome to minimize and mitigate its impact on metabolic health and QoL. The diagnosis is based on key clinical features, including hyperandrogenemia (manifesting as acne and hirsutism), IR, and AN, along with other associated metabolic abnormalities.

Effective management requires a multifaceted approach, with lifestyle modifications and weight loss as the cornerstone of treatment. Pharmacological interventions such as metformin, pioglitazone, and liraglutide play a crucial role in improving insulin sensitivity, while bariatric surgery may be an option for patients with severe obesity. Additionally, addressing the psychological burden through psychiatric support is essential for improving adherence to treatment and overall well-being.

In conclusion, a proactive, multidisciplinary approach is key to optimizing long-term outcomes in patients with HAIR-AN syndrome. Early intervention, patient education, and sustained treatment adherence are critical in mitigating complications and enhancing QoL.

## Figures and Tables

**Table 1:** Anthropometric measurements in years.

	2008	2011	2014	2017	2022
Age (years)	9	11	14	17	22
Height (cm)	165	167	169	172	172
Weight (kg)	84	93.5	90.2	104	118
BMI (body mass index)		33.3	31.3 (↑)	34.5 (↑)	40.4 (↑)
Waist (cm)			108	111	127
Hip (cm)			110	110	116
WHR (waist/hip ratio)			0.98 (↑)	1.01 (↑)	1.09 (↑)

**Table 2:** Laboratory results in years.

Date	2008	2011	2014	2017	2022
Glucose (mg/dL)	93	85	97	82	92
Insulin (uIU/mL)	21.5 (↑)	20.55 (↑)	32.3 (↑)	84.9 (↑)	30.4 (↑)
HOMA-IR	4.9	4.31	7.66	18.46	6.81
T-cholesterol (mg/dL)		164	203	211	218
Triglyceride (mg/dL)	212	107	110	288 (↑)	152
HDL (mg/dL)	39 (↓)	41.7 (↓)	44 (↓)	40 (↓)	39 (↓)
LDL (mg/dL)	114	100	121	113	149
AST (IU/L)	34	17	27	26	28
ALT (IU/L)	67 (↑)	36.8 (↑)	51 (↑)	71 (↑)	59 (↑)
GGT (U/L)				51 (↑)	60 (↑)
HgbA1c (%)		4.7			
TSH (mU/L)	2.9	2.5	4.2	2.74	3.2
FSH (mIU/mL)		4.48	7.1	4.69	3.67
LH (mIU/mL)		9.53	18.7 (↑)	7.08	3.97
E2 (pg/mL)			28.1	21.4	34.8
*t*-test (ng/mL) (0.06–0.82)		0.39	0.76	0.53	0.33
Cortisol (ug/dL)	8.04		26.1 (↑)	10.5	11.4
Prolactin (ng/mL)		12.5	8.9	18.5	12.3
GH (ng/mL)		0.2			0.2
DHEAS (ug/dL) (33–280)		359	460 (↑)	405 (↑)	329
SHBG (nmol/L)		12.4 (↓)		15.2 (↓)	22.1 (↓)
1 mg suppression	0.8			0.35	
Free androgen index (%0.5–7.3)		10.9		12.2	5.2
17 OH PG (ng/mL)				0.94	

## Data Availability

Data are available on request from the authors.
